# Glances at the Hospitals

**Published:** 1902-01-04

**Authors:** 


### GLANCES AT THE HOSPITALS.

### ABERDEEN ROYAL INFIRMARY.

THE total income was £11,708, and the total expenditure was £12,032, showing a deficit in the year's working of £323. It would seem that the income was more than £1,500 in excess of that of the previous year; but then more beds were occupied, and higher prices had to be given for most of the articles consumed during the year. It is not surprising that the cost per bed occupied rose from £48 to £50 13s. At the beginning of the year there were 199 patients in the infirmary. The admissions were 2,477, making a total of 2,646. Of these 1,201 recovered, 728 improved, 302 discharged for various reasons, 206 died, and 209 remained under treatment. The average number in the infirmary was 207, the highest 229, the lowest 190, and the average stay in the infirmary was 28½ days—an average which strikes us as being very high. The number of operations on in-patients was 906. The out-patients reached the respectable figure of 7,265. In the electrical department 126 patients were treated. Skiagrams were taken in 176 cases, and 316 received massage. Anæsthetics were administered 1,342 times. The medical and surgical tables are models of what these things should be.

### KILMARNOCK INFIRMARY.

THE total ordinary income for the year was £4,517, and the total ordinary expenditure was £4,681. The income was £442 less than in the previous year; but the workpeople's own subscriptions reached £816, being an increase of £25. It is not quite satisfactory that, with increasing work devolving on the hospital, caused by an ever-increasing population, the general subscriptions should show a tendency to decrease. Additions have been made to the infirmary at a cost of £1,250; and, although this has been paid for, the directors make a special appeal to the public to come to their aid and wipe off the adverse balance on the maintenance account. As this is only £429 it is hard to believe that the inhabitants will not speedily find the sum asked for. Owing to an outbreak of typhoid fever the institution was taxed to its utmost, and 264 patients were treated in the fever wards. At the beginning of the year there were in the medical, surgical, and fever wards a total of 111 patients, and altogether 1,044 were admitted during the year. Of those treated to a termination, 729 were cured, 169 were improved, 20 not improved, 5 were discharged for various reasons, and 89 died. There remained under treatment 126. The tables are very clearly compiled. Of 178 cases of scarlet fever, 148 recovered, 5 died, and 25 remained. Of 94 cases of enteric fever, 59 recovered, 8 died, and 27 remained.

